# Evaluation of the Potential of Sasobit REDUX Additive to Lower Warm-Mix Asphalt Production Temperature

**DOI:** 10.3390/ma12081285

**Published:** 2019-04-19

**Authors:** Arminda Almeida, Michela Sergio

**Affiliations:** 1 Department of Civil Engineering, University of Coimbra, 3030 Coimbra, Portugal; 2 CITTA Research Centre for Territory, Transports and Environment, 3030 Coimbra, Portugal; 3 Department of Civil, Construction and Environmental Engineering, La Sapienza University of Rome, 00185 Rome, Italy; sergio.1475665@studenti.uniroma1.it

**Keywords:** warm mix asphalt, Sasobit REDUX, temperature reduction, Marshall test, compactability test

## Abstract

Environmental and health concerns have been increasing in the road construction industry. This industry has provided several techniques and a wide range of additives to lower the production temperatures of asphalt mixtures, generating, among others, a new mix type called warm-mix asphalt (WMA). This paper aims to evaluate the potential of the Sasobit REDUX additive to lower the production temperatures of WMA. This additive, which is an alternative to the well-known Sasobit, is completely soluble in bitumen at temperatures above 85 °C while the same temperature for the Sasobit is 115 °C. For that reason, three target compaction temperatures were considered (90, 100 and 110 °C) and both Marshall and compactability tests were carried out. A hot-mix asphalt (HMA) was tested in parallel for comparison. It was concluded that the volumetric properties (air voids content about 4%) and the Marshall properties (stability about 11 kN, flow about 4 mm and Marshall quotient higher than 2 kN/km) of the Sasobit REDUX-WMA were globally satisfactory. In relation to the compactability test, the Sasobit REDUX-WMA mixtures were relatively easier to be compacted compared to the HMA mixture. The three Sasobit REDUX-WMA mixtures (90, 100 and 110 °C) exhibited a very similar compactability (differences lower than 0.4%). Therefore, it seems reasonable to conclude that the Sasobit REDUX has potential to lower WMA production temperatures by 20 °C. A reduction of that magnitude would lead to significant environmental gains.

## 1. Introduction

Asphalt mixtures are used worldwide to build roads. They are usually composed of aggregate, filler, and bitumen, and are manufactured in central plants. Once that mixture has been produced, it is transported to the road construction site for laying and compacting. Different technologies and processes can be applied and consequently different mixtures can be produced. Hot-mix asphalt (HMA) is the most produced one. However, environmental and health concerns have been increasing the use of techniques to produce and compact mixtures at lower temperatures. Warm-mix asphalt (WMA) results from one of those techniques. It is usually produced in a temperature range from 100 to 140 °C, while HMA needs production temperatures above 140 °C [1]. Comparing with HMA, WMA can provide a reduction of 24% on the air pollution impact and a reduction of 18% on fossil fuel consumption [2]. Mallick and Bergendahl [3] measured, in the laboratory using Dräger test equipment, CO_2_ emissions from the oxidation of bitumen in asphalt mixtures (HMA and WMA) with the purpose to analyze the effect of Sasobit, asphalt content and temperature on CO_2_ emissions. They found that temperature is the most important factor. Then, they developed regression models to estimate the reduction in CO_2_ emissions due to a decrease in temperature. Reductions greater than 30% were achieved and this percentage greatly increases when the production temperature of WMA decreases. Capitão, Picado-Santos and Martinho [1] presented a balance between the WMA-advantages and the WMA-drawbacks, which is next summarized: WMA reduces emissions, energy consumption, and the fume exposure of the workers. In addition, as the viscosity of the stiff binder decreases, the drop of temperature with time is less important, allowing higher haulage distances, reducing the risk of compaction troubles and the time to cool before opening it to traffic or to place the next layer. In contrast, additional costs to prepare the central plants and additives may not be compensated by the energy cost reduction. Obviously, these additional costs will depend to a great extent of the technique and additive used. 

Nowadays, several techniques and technologies exist to produce WMA. The most widely used classification differentiates them into three categories [4,5,6]: (i) foaming processes; (ii) addition of organic additives; and, (iii) addition of chemical additives. Foaming processes can be divided into water-based processes and water-bearing additives. In water-based processes, a small controlled amount of cold water is added to the bitumen via foaming nozzles resulting in a temporary expansion of its volume together with a reduction of its viscosity. In water-bearing additives, the foaming mechanisms in the asphalt binder are induced by using water-bearing additives such as synthetic zeolites (e.g., Aspha-min and Advera) or moistened fine aggregates (low energy asphalt—LEA, double barrel green, WAM foam, LEAB, ultrafoam GX, LT asphalt, low emission). A comprehensive review of foaming processes can be found in Reference [7]. In relation to the other two categories, the addition of organic and chemical additives, a wet process (incorporating the additive into the bitumen) or a dry one (incorporating the additive into the aggregates) can be performed. The organic additive (e.g., Sasobit and Asphaltan-B), when added to the mixture, melts changing the bitumen temperature-viscosity curve and consequently, the mixing and the compacting can be done at a relatively low temperature. The organic additive should be chosen in a way in which its melting point would be higher than pavement service temperatures since they can alter binder grade, increase high-temperature grade and decrease low-temperature grade [8]. Chemical additives (e.g., Cecabase, Evotherm-ET, DAT, 3G, Rediset), in turn, improve coating, mixture workability, and compaction.

Depending on the techniques and technologies different reductions on production temperature can be achieved [5]. Foaming processes are most popular but additives are gaining market share since additives are easy to use, leads with higher temperature reductions, alter binder grade, have anti-strip properties and plant modifications are not required (they can be added to the binder at the asphalt terminal or in the plant supply tank, or they can be added to the mixture by blowing them into the drum). Studies comparing techniques and technologies can be found in the literature [5,9]. 

Driven essentially by a reduction in environmental impact comparing HMA, the use of WMA has been increasing. In 2017, with 38.7% of the market share, the USA was the world’s largest producer of WMA [10]. The European champion was France, with a market share of 11.4%, followed by Norway (11.1%) and Denmark (8.5%). These differences in market share between the USA and Europe can be justified by the mix asphalt plant they often use, counter flow plants in the USA against batch plants in Europe [11].

This paper uses a synthetic (Fischer-Tropsch process) paraffin wax additive (organic additive technology), more precisely the Sasobit REDUX, which is an alternative to the well-known additive Sasobit. The Sasobit-REDUX was introduced in June 2016, and there are very few references in the literature addressing it. In the b-on database, just one paper appeared on 1 March 2019 with the “Sasobit REDUX” search term [12]. In that paper, bitumen modification with 3% of Sasobit REDUX was studied. The Sasobit, in turn, was introduced 20 years ago to the asphalt industry [13]. Edwards and Isacsson [8,14] carried out an extensive literature review concerning wax in bitumen, pointed, among other things, the crystallinity and the melting properties of the wax as important factors. At higher temperatures, melted waxes may decrease the WMA resistance to rutting while, at low temperatures, wax crystallization can lead to mixture cracking [15]. The effect of wax in bitumen depends greatly on its content. Sasobit contents higher than 4% affect negatively the low-temperature properties of the bitumen [14]. For this reason, contents higher than 3% are not recommended. Fazaeli, et al. [16] studied the performance of Sasobit-modified bitumen at high, intermediate and low temperatures using different Sasobit contents (1.0%, 2.0%, 2.5%, 3.0% and 4.0%). The modification improved the bitumen performance at high temperatures and did not show a considerable influence on the coldest temperatures. Wu, et al. [17] studied the top-down cracking field performance of WMA and compared it with similar HMA using 28 pavement projects covering different climate zones (dry freeze, wet freeze, dry no-freeze and wet no-freeze), WMA technologies (including the Sasobit wax), service years, pavement structures and traffic volume levels. They found that the exhibited performance of HMA and WMA is in general comparable. Only in one HMA-WMA pair (with Sasobit wax), the performance of the HMA was higher (wet-freeze zone). The authors pointed factors such as variations of the pavement structure, pre-overlay conditions, etc., as possible reasons. Chen, et al. [18] reviewed successfully, among other techniques and materials, the use of Sasobit-WMA in wet-freeze climate.

Jamshidi, et al. [19] summarized more than 230 pieces of literature on binder and WMA incorporating Sasobit. They present the effects of Sasobit on asphalt binder as well as the performance of WMA produced with it. Concerning the performance, aspects related to mix design, volumetric properties and engineering properties (rutting, fatigue and moisture sensitivity) are reviewed. In general, WMA presents a superior performance in terms of fatigue life. Laboratory studies have shown that the performance of Sasobit-WMA mixtures depends, among other factors, on mixing and compaction temperature, and Sasobit dosage. In general, the mechanical performance of WMA is satisfactory and similar to the one obtained with HMA. Due to lower production temperatures, moisture sensitivity of WMA has been studied. Xu, Xiao, Amirkhanian and Singh [6] carried out a review of the literature to identify the factors influencing the moisture characteristics. The following ones were identified: materials, technologies and compaction temperature. Looking only for Sasobit-WMA reported results against a control mixture (a total of nine studies), in two studies the tensile strength ratio (TSR) of the WMA was lower in more than 10%, in four studies the TSR of the WMA was just slightly lower and in the remaining three studies the TSR of the WMA was higher. Yu, et al. [20] also did not observe differences in moisture sensitivity between the Sasobit-WMA and the control mixture.

In contrast to Sasobit, which crystallizes from approximately 90 °C, forms a lattice structure in the bitumen and therefore increases significantly the stiffness of the bitumen, Sasobit REDUX crystallizes from approx. 60 °C and does not significantly change bitumen stiffness as the stiffness of Sasobit REDUX is in a similar range like the bitumen [13]. The Sasobit Redux is completely soluble in bitumen at temperatures above 85 °C while the same temperature for the Sasobit is 115 °C [13]. Therefore, the use of Sasobit REDUX allows a higher reduction in mixing and compaction temperatures. Meanwhile, despite the great amount of research regarding the use of Sasobit on WMA, there are a limited number of studies that consider the Sasobit REDUX. An important issue for the success of WMA technology is the definition of production temperatures, compactability tests are usually carried out [21,22,23]. Some studies prepare specimens using the Marshall impact compactor while others use the Superpave gyratory compactor (SGC). With the SGC, compaction is achieved by simultaneous action of low static compression and a shearing action. Therefore, it simulates the field conditions in a better way than the impact compactor does [24]. Compaction with SGC has also been considered to be one of the best methods of laboratory compaction for the assessment of compactability of asphalt mixtures [22].

This paper evaluates the potential of the Sasobit REDUX additive to lower the production temperatures of WMA. For that, the following three compaction temperatures are considered: 90, 100 and 110 °C, which are lower than the ones usually used with the Sasobit additive (110–120 °C), and Marshall tests and compactability tests using the SGC are carried out. In addition, the situation of a higher haulage distance is considered by mixing the Sasobit REDUX-WMA at 165 °C and compacting it at 110 °C. This paper constitutes thus a contribution to the state-of-the-art. 

## 2. Methodology and Materials

Two tests were considered in this research, Marshall compression tests to check the fulfilment of the Portuguese specifications [25] and to predict performance, and compactability tests to define production temperatures correctly.

**Figure 1:**
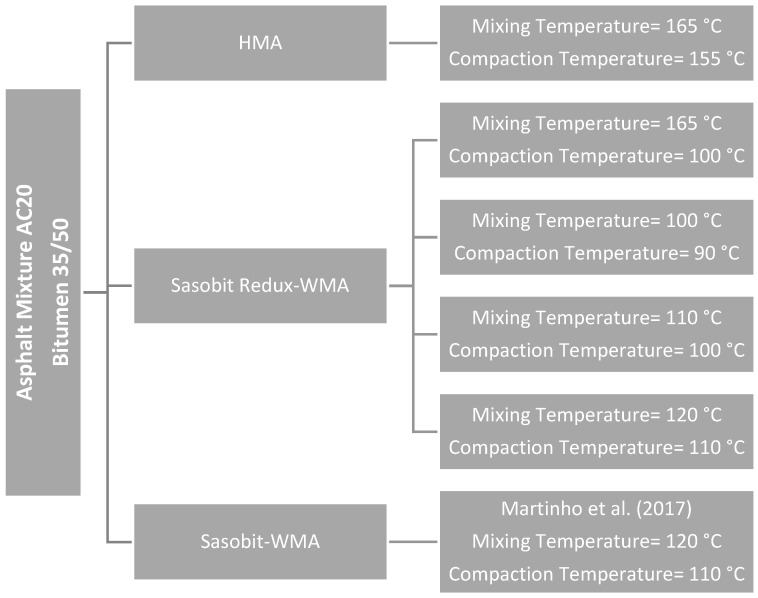
Asphalt mixtures and temperatures.

An asphalt mixture with a maximum aggregate size of 20 mm was selected (AC20), which is used in base layers. An HMA was also tested to use as a reference. Concerning a WMA with the Sasobit additive, results from Martinho, et al. [26] were considered. Figure 1 depicts the asphalt mixtures considered as well as the corresponding production temperatures (mixing and compaction). Besides lower production temperatures, the situation of higher haulage distance was considered for the Sasobit REDUX-WMA (mixing at 165 °C and compaction at 110 °C).

**Figure 2:**
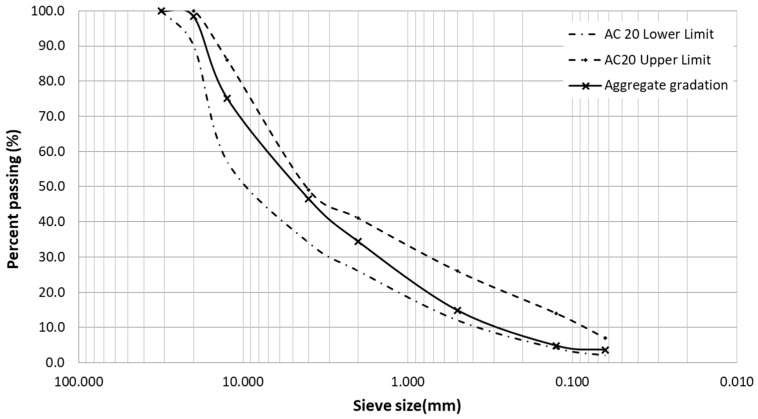
Aggregate gradation of the AC 20 mixture.

All the mixtures were produced with limestone aggregates and 35/50 paving grade bitumen. The binder content considered in this study was 5.0% by the weight of the mixture. Martinho, Picado-Santos and Capitão [26] considered 4.5%. The gradation limits used for the AC 20 mixture are defined in the Portuguese road administration specifications [25]. Figure 2 presents the aggregate gradation.

**Figure 3:**
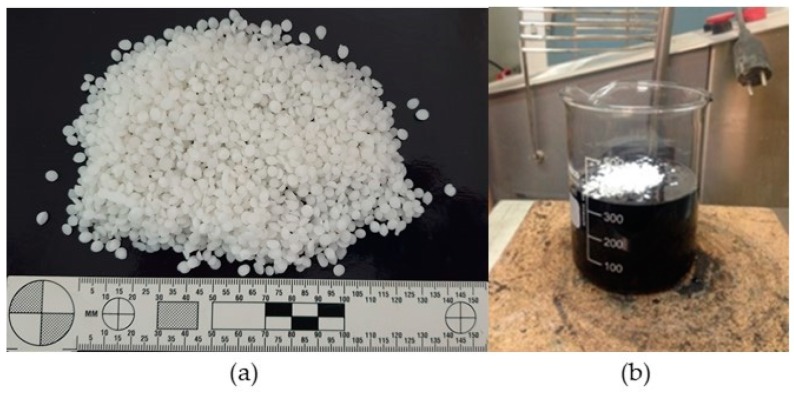
Sasobit REDUX (**a**) pastilles, (**b**) addition to bitumen.

The dosage of Sasobit REDUX used in this study was 1.5% by weight of bitumen, which was added to the bitumen and stirred until dissolved. Martinho, Picado-Santos and Capitão [26] considered a 4.0% of Sasobit. Table 1 compares the specifications of both additives. Figure 3 shows the Sasobit REDUX pastilles.

## 3. Specimen Preparation and Tests

### 3.1. Specimen Preparation

Marshall cylindrical samples of asphalt mixture (101.6 mm diameter and 63.5 mm height) were prepared using the impact compactor according to EN 12697-30 [27] applying 75 blows on each side of the cylindrical sample. Gyratory compaction cylindrical samples of asphalt mixture (150 mm diameter and 115 height) were compacted using the SGC according to EN 12697-31 [28] with a compaction pressure of 600 kPa, a dynamic angle of 1.25° (corresponding to an internal dynamic angle of 1.16°) and a rotation speed of 30 rpm. Both the mixing and the compaction temperatures were the ones defined in Figure 1.

### 3.2. Volumetric Properties 

So that asphalt mixtures can be applied on road pavements, their volumetric properties should meet certain limits. Regarding air voids content, the mixture must have sufficient voids in the total compacted mixture to allow for a slight amount of bitumen expansion due to temperature increases (without flushing, bleeding and loss of stability), but not so much to limit the permeability of harmful air and moisture into the mixture and the layers below [29]. For that, the bulk density of the mixtures as well as the theoretical maximum density were determined following the EN 12697-6 [30] and the EN 12697-5 [31], respectively. Then, the following volumetric properties: air voids content, voids in mineral aggregates (VMA) and voids filled with a binder (VFB) were evaluated according to the EN 12697-8 [32].

### 3.3. Marshall Test

The Marshall method is an empirical mix design method used in many European countries [33]. It is based on the Marshall test described in EN 12697-34 [32] that involves the application of a compressive load through semicircular testing heads and the determination of the stability and the flow properties. These properties provide a performance prediction of the mixture. The Marshall quotient was then calculated by dividing the Marshall stability by the flow. This Marshall quotient is a key index to the mixture stiffness [34,35].

### 3.4. Compactability Test

The compactability of an asphalt mixture can be defined as the ease with which the material can be compacted [36]. The EN 12697-10 [36] defines methods to characterize the compactability of asphalt mixtures by the relation between its density or voids content and the compaction energy applied to it. Since gyratory compaction is considered one of the best laboratory compaction methods for the assessment of compactability mixtures [22], it is used in this study. Therefore, the compaction curve depicts the degree of compaction (defined as the percentage of the maximum theoretical specific gravity of the compacted mixture) against the number of gyrations (plotted on a logarithmic scale). The EN 12697-10 proposes the Equation (1) to express the variation of the air voids content of the compacted specimen as a function of the compaction energy.

(1) $$V_{i}=V_{1}-K\times \ln\left(N_{i}\right),$$

where V_i_ is the air voids content for a given number of cycles (%), V_1_ is the air voids content calculated at the first gyration; K is the compactability factor, and N_i_ is the number of gyrations.

The relation between the air voids content and the degree of compaction (*C_i_*) for a given number of cycles is expressed by Equation (2).

(2) $$V_{i}=100-C_{i},$$

Therefore, Equation (1) can be rewritten using the degree of compaction instead of air voids content—Equation (3), where C_1_ is the degree of compaction at the first gyration.

(3) $$C_{i}=C_{1}+K\times \ln\left(N_{i}\right).$$

Equation (3) was used to fit the gyratory compaction curves. The parameters *C_1_* and *K* represent the self-compaction and the workability of the mixtures, respectively, and they are usually used to quantify the compaction properties and to compare different mixtures. The self-compaction parameter is related with the proneness of the mixture to be compacted under the paver during laying operations and the workability parameter (K) is related with the ability of the mixture to achieve a stable stone-to-stone contact under the rollers during the compaction process [37]. This procedure was also used by Mo, Li, Fang, Huurman and Wu [22] to investigate the effect of temperature of a WMA containing chemical additives.

## 4. Results and Discussion

### 4.1. Volumetric Properties

**Figure 4:**
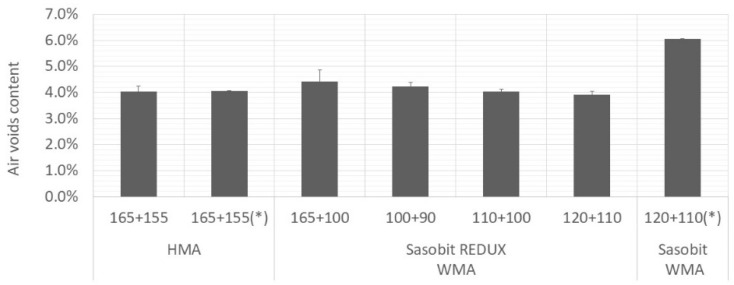
Variation of air voids content with mixture and temperature.

**Figure 5:**
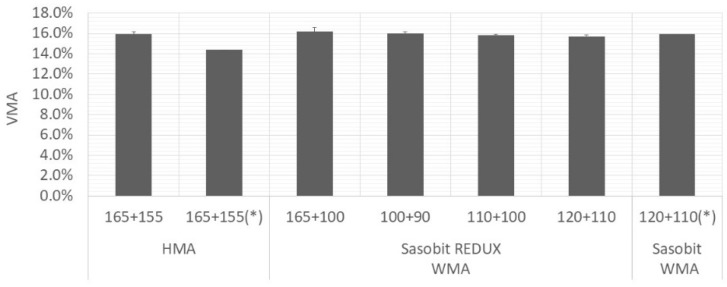
Variation of VMA with mixture and temperature.

**Figure 6:**
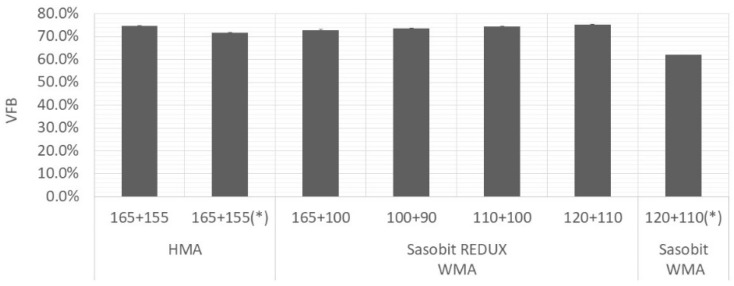
Variation of VFB with mixture and temperature.

Figure 4, Figure 5 and Figure 6 present average values of air voids content, VMA and VFB, respectively. The vertical error bar represents the standard deviation of four specimens and the asterisk means that are values from Martinho, Picado-Santos and Capitão [26].

As expected, the air voids content increased with decrease in production temperatures. All the mixtures, even the Sasobit REDUX-WMA compacted at 90 °C, fulfil the limits imposed by Portuguese specifications [25]. For the mixture under study (AC20 base), the air voids content should be in the range 3%–6% and the VMA should be at least 14%. It will allow bitumen expansion and at the same time, it will limit the permeability of harmful air and moisture [29].

### 4.2. Marshall Test

**Figure 7:**
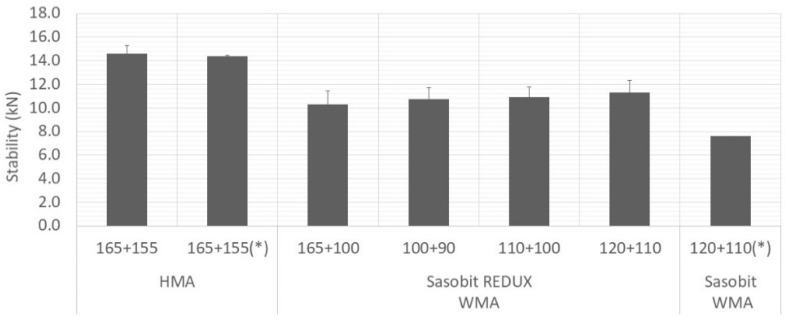
Variation of Marshall stability with mixture and temperature.

**Figure 8:**
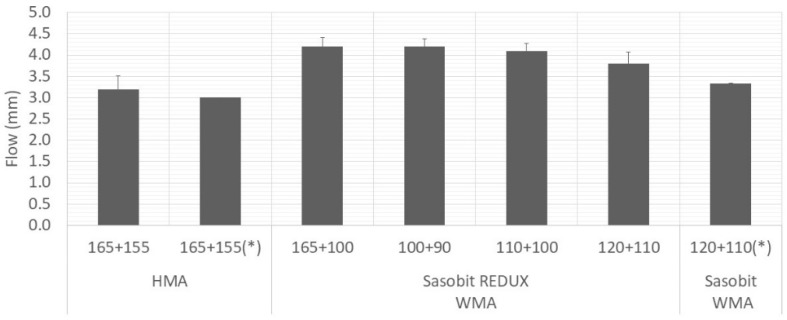
Variation of Marshall flow with mixture and temperature.

**Figure 9:**
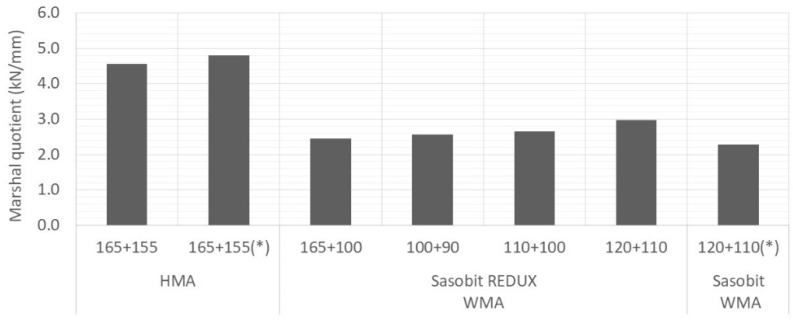
Variation of Marshall quotient with mixture and temperature.

Figure 7, Figure 8 and Figure 9 show average values obtained for stability, flow and Marshall quotient, respectively. Again, the vertical error bar represents the standard deviation of four specimens and the asterisk means that are values from Martinho, Picado-Santos and Capitão [26].

Portuguese specifications impose limits on the values of these properties. For the mixture under study (AC20), the stability must be in the range 7.5–15 kN, the flow in the range 2–4 mm and the Marshall quotient should be at least 2 kN/mm [25]. The WMA yielded stability values lower than the HMA but above the minimum limit (7.5 kN). In relation to the flow, some values are just above the upper limit (4 mm) and above the ones obtained using the additive Sasobit [26], which may be explained by the bitumen dosage used, 5.0% against the 4.5% used in Martinho, Picado-Santos and Capitão [26]. Nevertheless, the Marshall quotients are all higher than 2 kN/mm. 

For the three compaction temperatures tested (90, 100 and 110 °C), the results were very similar. Therefore, looking for the volumetric and the Marshall properties, the Sasobit REDUX additive has the potential to lower the production temperature of WMA at 20 °C. 

### 4.3. Compactability Test

**Figure 10:**
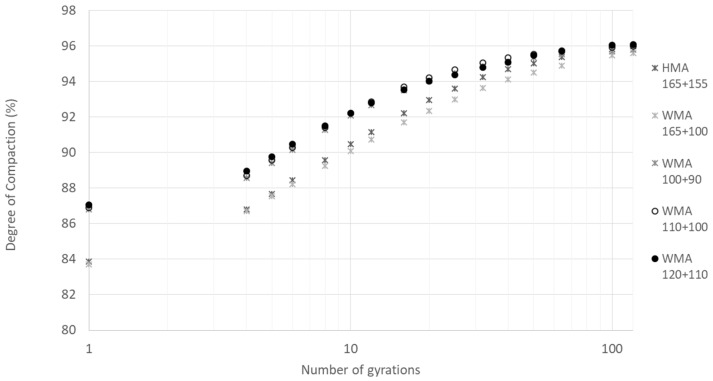
Gyratory compaction curves for the tested mixtures and temperatures.

**Figure 11:**
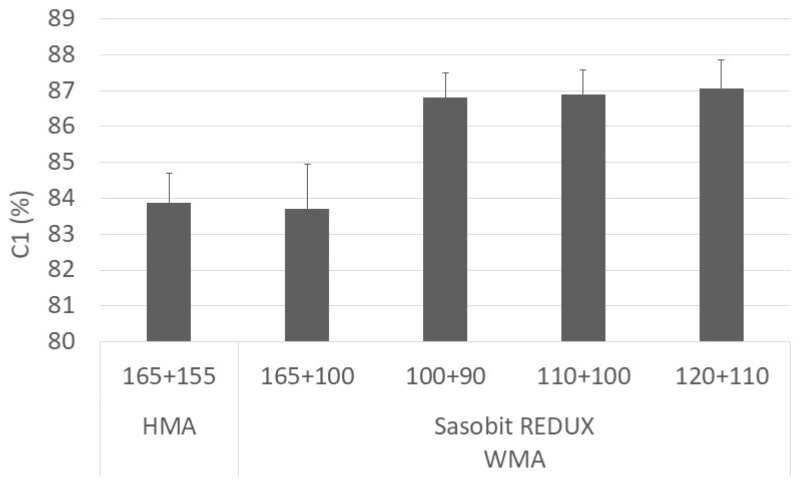
Variation of C1 parameter with mixture and temperature.

**Figure 12:**
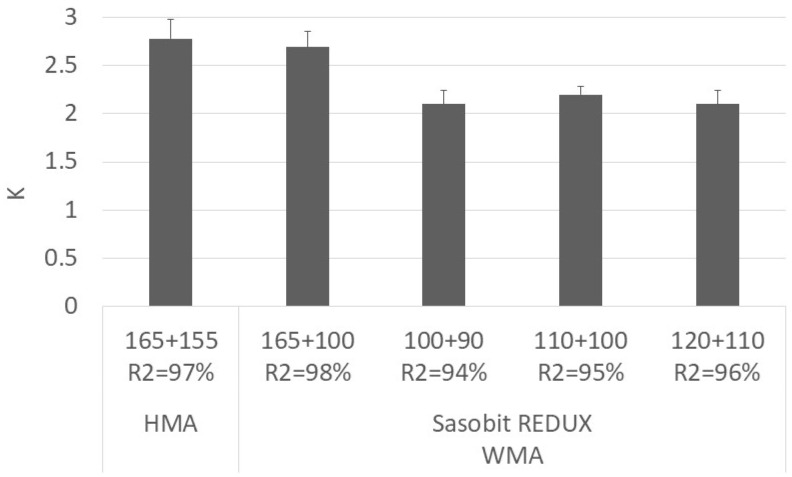
Variation of K parameter with mixture and temperature and corresponding squared-correlation values.

Figure 10 presents the development of the degree of compaction with the number of gyrations. The parameters, C_1_ and K, obtained by fitting Equation (3) to the compaction curves are presented in Figure 11 and Figure 12, respectively. The vertical error bar represents the standard deviation of four specimens. In Figure 12, it is also possible to see the squared-correlation values which are in the range 94%–98%.

From the figures, it is possible to separate the mixtures into two sets, the ones produced at a higher temperature (mixing at 165 °C) and the others produced at lower temperatures. The first set includes the HMA and the WMA produced at 165 °C and transported for a long distance (higher haulage distance). The mixtures on the second set (WMA produced at lower temperatures) presents better compaction than the mixtures on the first set (HMA and WMA for higher haulage distances). Concerning the WMA mixtures produced at lower temperatures, the variation of the degree of compaction at a specific number of gyrations is limited to 0.4%. It indicates that the compactability of the mixture compacted at 90 °C is equal to the one compacted at 110 °C.

The HMA results are in line with those reported by Gardete, et al. [38] who also tested an AC 20 mixture (35/50 paving grade bitumen) considering different bitumen contents. For a bitumen content of 5.0%, the K value was 2.7 and the air voids content at the first gyration was 16%.

## 5. Conclusions

Marshall and compactability tests were conducted to evaluate the potential of the Sasobit REDUX additive to lower the production temperatures of WMA. This additive, which is an alternative to the well-known Sasobit, is completely soluble in bitumen at temperatures above 85 °C while the same temperature for the well-known Sasobit is 115 °C. Three target compaction temperatures were considered (90, 100 and 110 °C) as well as the situation of higher haulage distance. An HMA with the same bitumen and aggregates was tested and used as a reference.

Based on the results presents in the text the following conclusions can be drawn:

- Although the WMA-Marshall properties were inferior to the HMA ones, the Marshall properties of the Sasobit REDUX-WMA fulfilled the specifications and they were then globally satisfactory.
- The Sasobit REDUX-Marshall stability was higher than the Sasobit one.
- The compactability of the Sasobit REDUX-WMA produced at lower production temperatures was better than the achieved with the HMA.
- The compactability of the Sasobit REDUX-WMA compacted at 90 °C is equal to the one compacted at 110 °C. Therefore, the compaction temperature of the Sasobit REDUX-WMA has the potential to be reduced by 20 °C. Such a reduction in production temperatures would have important environmental benefits. Besides energy cost reduction, this technology leads to a decrease in the emissions of gases and odors from asphalt plants, and an improvement in the personal working conditions.

The presented research evaluated successfully the potential of the Sasobit REDUX additive to lower WMA production temperatures. This is thus a starting point for performance evaluation which should encompass the determination of stiffness, rutting resistance, fatigue resistance and moisture susceptibility.

## Figures and Tables

**Table 1:** Comparison of Sasobit Redux and Sasobit specifications [13].

Parameter	Sasobit REDUX	Sasobit
Melting point (°C)	72–83	100–110
Penetration (25 °C)—0.1 mm	16–30	0–20
